# Rapid Implementation of Video Visits in Neurology During COVID-19: Mixed Methods Evaluation

**DOI:** 10.2196/24328

**Published:** 2020-12-09

**Authors:** Erika A Saliba-Gustafsson, Rebecca Miller-Kuhlmann, Samantha M R Kling, Donn W Garvert, Cati G Brown-Johnson, Anna Sophia Lestoquoy, Mae-Richelle Verano, Laurice Yang, Jessica Falco-Walter, Jonathan G Shaw, Steven M Asch, Carl A Gold, Marcy Winget

**Affiliations:** 1 Primary Care and Population Health Stanford University School of Medicine Stanford University Palo Alto, CA United States; 2 Department of Neurology & Neurological Sciences Stanford University School of Medicine Stanford University Palo Alto, CA United States; 3 Center for Innovation to Implementation (Ci2i) VA Palo Alto Health Care System Menlo Park, CA United States

**Keywords:** teleneurology, telemedicine, telehealth, ambulatory neurology, video visits, COVID-19, implementation, outcomes, video, neurology, mixed methods, acceptability, sustainability

## Abstract

**Background:**

Telemedicine has been used for decades. Despite its many advantages, its uptake and rigorous evaluation of feasibility across neurology’s ambulatory subspecialties has been sparse. However, the COVID-19 pandemic prompted health care systems worldwide to reconsider traditional health care delivery. To safeguard health care workers and patients, many health care systems quickly transitioned to telemedicine, including across neurology subspecialties, providing a new opportunity to evaluate this modality of care.

**Objective:**

To evaluate the accelerated implementation of video visits in ambulatory neurology during the COVID-19 pandemic, we used mixed methods to assess adoption, acceptability, appropriateness, and perceptions of potential sustainability.

**Methods:**

Video visits were launched rapidly in ambulatory neurology clinics of a large academic medical center. To assess adoption, we analyzed clinician-level scheduling data collected between March 22 and May 16, 2020. We assessed acceptability, appropriateness, and sustainability via a clinician survey (n=48) and semistructured interviews with providers (n=30) completed between March and May 2020.

**Results:**

Video visits were adopted rapidly; overall, 65 (98%) clinicians integrated video visits into their workflow within the first 6 implementation weeks and 92% of all visits were conducted via video. Video visits were largely considered acceptable by clinicians, although various technological issues impacted their satisfaction. Video visits were reported to be more convenient for patients, families, and caregivers than in-person visits; however, access to technology, the patient’s technological capacity, and language difficulties were considered barriers. Many clinicians expressed optimism about future utilization of video visits in neurology. They believed that video visits promote continuity of care and can be incorporated into their practice long-term, although several insisted that they can never replace the in-person examination.

**Conclusions:**

Video visits are an important addition to clinical care in ambulatory neurology and are anticipated to remain a permanent supplement to in-person visits, promoting patient care continuity, and flexibility for patients and clinicians alike.

## Introduction

Telemedicine has been used for decades, yet its uptake and rigorous evaluation of feasibility across neurology’s ambulatory subspecialties has been sparse [1,2]. Studies have shown several advantages of telemedicine including improved access to care [3,4], less travel burden, and fewer associated out-of-pocket and health care costs [1,5-8]. Nonetheless, integration of virtual visits into ambulatory neurology care, where the physical examination and nuanced communication play a strong role, has been received with some hesitation [7,9-13]. Further, issues related to technology, compensation, payor reimbursement, policy, hardware/software costs, credentialing, liability, and requirements for in-person evaluations prior to virtual care, have also limited its adoption [1,8,11,13-17].

The declaration of COVID-19 as a pandemic in March 2020 prompted health care systems worldwide to reconsider traditional health care delivery and quickly transition to telemedicine [18-21]. In regions with rapidly increasing COVID-19 cases and early stay-at-home directives, health care systems rapidly built and implemented infrastructure for telemedicine technologies to protect health care workers and patients, and conserve personal protective equipment. To further support this pivot and maintain health care access, the United States loosened previously stringent federal regulations on reimbursements, licensing, and Health Insurance Portability and Accountability Act (HIPAA) compliance [22,23]. In neurology specifically, the urgent need to provide care safely to patients with chronic illnesses while preventing disease transmission during clinic visits, led to rapid implementation of video visits across all subspecialties [24-29].

In this study, we evaluate the implementation of video visits in Stanford Health Care’s (California, United States) ambulatory neurology clinics using mixed methods to assess adoption and explore clinicians’ perspectives on the acceptability, appropriateness, and sustainability of this broad expansion.

## Methods

### Setting

This quality improvement project received a nonresearch determination by Stanford University’s Institutional Review Board (IRB-55644). It was conducted at Stanford University’s Department of Neurology and Neurological Sciences, which includes 11 ambulatory subspecialties staffed by 60 physicians and 8 advanced practice providers (APPs). As previously described [27], at the beginning of the local COVID-19 stay-at-home directive in March 2020, approximately 50 in-clinic computers were video visit–enabled, and 50 additional computer devices were readied for remote use by providers. Clinic staff converted >90% of scheduled in-person visits to video. In-person visits were reserved for procedures such as autonomic testing, implanted device programming, injections, electromyography, and electroencephalograms, as well as patients determined either during previsit screening or initial video visit to require a full examination.

### Primary Outcomes, Data Collection, and Analysis

#### Overview

The primary implementation outcomes assessed were adoption, acceptability, appropriateness, and perceived sustainability of video visits [30], as described in Table 1. Clinician-level scheduling data were used to assess adoption. A combination of a clinician survey and interviews were used to assess acceptability, appropriateness, and perceived sustainability. Clinicians included physicians and APPs.

**Table 1:** Implementation outcomes, definitions, and data sources applied in the evaluation of the implementation of video visits in ambulatory neurology.

Proctor et al’s [30] implementation outcomes	Definitions	Data sources
Adoption	Uptake of video visits in ambulatory neurology	Scheduling data
Acceptability	Clinicians’ overall satisfaction with video visits	Clinician interviews and clinician survey
Appropriateness	“Fit for purpose”: clinicians’ perceived suitability and practicability of video visits for a successful patient visit to achieve similar patient outcomes to an in-person visit	Clinician interviews and clinician survey
Sustainability	Clinicians’ views on the future use of video visits in their practice	Clinician interviews and clinician survey

#### Scheduling Data

Clinician-level scheduling data were extracted for all clinicians who had the opportunity to conduct video visits. The first week of the stay-at-home order (March 15-21, 2020) was considered a transition week where the physician champion onboarded physicians onto the video visit platform. To assess our primary outcome, adoption, scheduling data from the 8-week implementation period (March 22 through May 16, 2020) was used for analysis; the transition week was excluded. Adoption was assessed in three ways: (1) proportion of clinicians who conducted video visits, (2) proportion of visits completed via video during the COVID-19 pandemic, and (3) proportion of all “expected” visits that were done via video, where “expected” was defined as the estimated number of visits completed had COVID-19 not occurred (ie, visit volume from the same calendar period in the prior year, March 24 through May 18, 2019).

The number of visits completed during the implementation period (ie, numerator) and comparator period (ie, denominator) included visit types that could be feasibly conducted in-person or via video. Visit types inherently requiring in-person patient interaction, such as procedures and research visits, were excluded. The percentage of “expected” visits is shown for video and in-person visits separately, along with the percentage of lost potential visits. The proportion of all “expected” visits completed is a measure of the proportion of “saved” or “protected” visits attributable to video visits during the COVID-19 stay-at-home period*.*

#### Clinician Survey

We developed a 20-item survey informed by early interview findings and reflections from the clinical improvement team. The survey was administered via Stanford’s REDCap platform [31] and emailed to all clinicians using video visits in May 2020, followed by two reminders. To increase response rates, clinicians were encouraged to complete the survey during division meetings and reminded personally by clinical leaders. Descriptive analyses were conducted on 5 items (Multimedia Appendix 1): (1) need for and timing of in-person visits to supplement video visits, (2) top three concerns regarding video visits, (3) top three reasons for excitement for video visits, (4) video visits support of overall clinician wellbeing, and (5) video visit usage to shift uncompensated to compensated work. The number and percentage of clinicians indicating each response are reported for questions 1-3 and for those indicating “agree” or “strongly agree” for questions 4 and 5.

All quantitative data were processed and analyzed using SAS (Version 9.4; SAS Institute Inc), R (Version 4.0.2; R Foundation for Statistical Computing) [32] and associated packages [33-36].

#### Clinician Interviews

We designed the interview guide (Multimedia Appendix 2) to encompass three implementation outcomes of interest: acceptability, appropriateness, and perceived sustainability [30]. A purposive sample of 47 clinicians who had conducted at least one video visit were invited to participate in semistructured phone interviews held between March and May 2020. Stakeholders were intentionally recruited from different neurology subspecialties to obtain a representative departmental sample. In total, 30 clinicians were interviewed and interviews lasted 25 minutes, on average.

Interview notes and transcripts were used for analysis. To ensure anonymity, identifiable information was removed from transcripts and subspecialties with <5 clinicians were grouped. Data were analyzed using both deductive and inductive approaches. Deductive codes were derived from the implementation outcomes of interest [30]. During analysis, barriers, facilitators, and other emergent themes were identified and coded. A multiphase analysis approach leveraged rapid analytic procedures (eg, template summaries) to extract early themes, consensus coding of transcript summaries to produce interim results, and a matrix analysis [37] of interview excerpts for final comparison of themes across neurology subspecialties. First, individual interview transcripts were summarized independently by two coauthors into a templated summary document. After review and consensus discussion, transcript summaries were consolidated into a matrix to identify themes and allow for comparison across participants using Microsoft Excel (Microsoft Corp). Four qualitative coauthors met weekly for two months to discuss preliminary results and achieve reporting consensus.

#### Mixed Methods Analysis

Quantitative and qualitative data were consolidated during analysis and interpreted in parallel to understand the impact of video visits in ambulatory neurology more comprehensively. This approach allowed us to harness strengths and offset weaknesses of the two methodologies [38,39]. We were also able to identify converging and diverging issues regarding adoption, acceptability, appropriateness, and perceived sustainability of video visits.

## Results

### Participants

Out of the 68 clinicians in the department’s 11 ambulatory subspecialties, 66 clinicians conducted regular video visits during the 8-week implementation period and were included in analyses (two clinicians were on leave and thus excluded). Table 2 summarizes the number of surveyed and interviewed clinicians, by subspecialty. In total, 48 (73%) clinicians responded to the survey and 30 (45%) were interviewed. In total, 21 (32%) clinicians participated in both the survey and interviews. Only 9 (14%) did not participate in either modality.

**Table 2:** Number of ambulatory neurology clinicians who completed the video visit survey and were interviewed, by subspecialty.

Subspecialty	Interview respondents, n	Survey respondents, n	Total clinicians, n
Autonomic, neuro-oncology, and neuro-ophthalmology^a^	3	4	9
Epilepsy	4	9	10
General neurology	2	5	5
Headache	4	3	5
Memory	3	4	6
Movement disorders	3	8	9
Neuroimmunology	3	5	5
Neuromuscular	4	5	8
Stroke	4	5	9
Total	30 (45%)^b^	48 (73%)^c^	66 (100%)

^a^The autonomic, neuro-oncology, and neuro-ophthalmology subspecialties had <5 clinicians and were therefore grouped to ensure anonymity.

^b^Of the 30 interviewees, 29 were physicians and 1 was an advanced practice provider.

^c^Of the 48 survey respondents, 41 were physicians and 7 were advanced practice providers.

### Adoption

**Figure 1:**
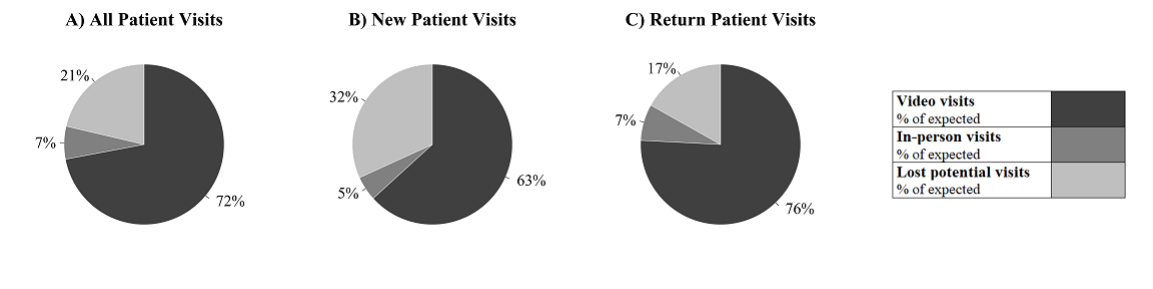
Percentage of video visits conducted between March 22 and May 16, 2020, as a proportion of (A) all "expected" visits overall, (B) "expected" new patient visits, and (C) "expected" return patient visits. The "expected" number of visits is defined as the number of completed visits in the comparable 2019 time period. Data only reflect operational scheduling data captured through the EPIC Hyperspace Platform. The data do not reflect no shows, visits conducted on other HIPAA-compliant software or visits that reverted to phone calls. (HIPAA: Health Insurance Portability and Accountability Act).

During the transition week, the clinician champion onboarded 30 of the 66 physicians included in this study, and 129 video visits were conducted. Prior to this transition week, clinicians in these ambulatory neurology clinics had not used video visits. During the 8-week implementation period, video visit adoption was high based on both percentage of clinicians using them, and percentage of visits completed via video. Within the first two weeks, 52 (79%) clinicians integrated video into their practice, increasing to 65 (98%) clinicians by week 6. Almost all (92%) visits were conducted via video and adoption of video visits was high for both new and return patient visits (93% and 91% of completed visits, respectively). The total number of completed visits, conservatively estimated, was almost 80% of expected visits based on the 2019 comparison period (Figure 1A). Clinics recuperated more expected return patient (83%) than new patient visit volumes (68%; Figures 1B-C).

### Acceptability

Overall acceptability of video visits was high, but clinicians experienced technological and scheduling issues, and identified additional needs. Two themes emerged related to acceptance: technology and workflow efficiency.

#### Technology

While some clinicians experienced a smooth transition to video visits and seamless connection with patients, others dealt with numerous technical issues, jeopardizing their overall satisfaction. Indeed, technology was one of the top 3 concerns regarding video visits for 30 (63%) survey respondents (Table 3).

**Table 3:** Ambulatory neurology clinicians’ (n=48) top 3 areas of concern related to video visits as reported through survey data.

With regard to video visits, clinicians (n=48) were concerned about... (indicated top 3)	Survey respondents, n (%)
Technological limitations	30 (63)
Being able to engage in training and education of residents and fellows	21 (44)
Missing/losing the in-person connection/relationship with patients	18 (38)
Including interpreters on video calls	17 (35)
Difficulties arranging and completing necessary follow-ups after the video visit	14 (29)
Insurance reimbursements for video visits are not the same as for in-person visits	8 (17)
Patients’ unwillingness to come into clinic for requested in-person visits in the future	7 (15)
Press-Ganey scores	5 (10)
Patient expectations to have video visits as an option	3 (6)
Maintaining access to readily available technology and equipment needed for video visits	2 (4)
Other (providers listed various reasons)	9 (19)

Several video functionalities were suggested for a successful visit, including the following: (1) *screen sharing* to facilitate patient education and explain imaging results, (2) a *waiting room function* to replicate “stepping out of the room” when engaging with trainees, (3) a *chat box* for troubleshooting, (4) *file sharing* capabilities, (5) *screenshot* capabilities to support efficient charting, and (6) *multiperson teleconferencing* to include other members of the multidisciplinary team, interpreters, trainees, and family members in different physical locations. These functionalities were not available during the study period.

...when we're discussing end-of-life care issues, to have the physician and respiratory therapist, social worker and palliative care physician, all present in the same ‘virtual room’, that would be really nice.

MD19

Engaging in education of trainees was one of the top 3 concerns for 21 (44%) surveyed clinicians (Table 3). The initial interface available through the electronic medical record (EMR) only allowed for a two-way call; therefore, parallel software was needed to include trainees.

#### Workflow Efficiency

Perceptions of workflow efficiency were mixed. Major issues included rigid video visit scheduling, note-taking efficiency, and previsit planning. Several clinicians mentioned that, provided there were no technological issues, video visits helped them stay on schedule. However, hard time limits set by the video visit platform caused frustration. Clinicians could not notify the next patient when running late nor initiate the next visit earlier than scheduled as they might do at the clinic.

…it's much easier for someone to wait in a physical waiting room if you do happen to run over with a patient who’s before them. It’s a lot harder or less acceptable if someone is waiting to see you via video and staring at their computer screen for 10 or 15 minutes. … You can’t open up another video and say, ‘I'm here. I'm just with somebody else, and I'm going to switch back.’

MD10

Views on documentation during a video call also differed. Note-taking was considered easier by some during video visits, reducing after-hours charting time. Others were unable to document simultaneously, making them less efficient. Dictation was considered a possible solution.

…unless you get the screen set up correct[ly], it actually can be really hard to document and type while doing the video visit. The typing can be noisy on the patient’s end…

MD5

However, most surveyed clinicians (n=34; 71%) agreed or strongly agreed that video visits allowed them to shift uncompensated to compensated work (ie, scheduling a video visit to address concerns that would previously have been managed through either EMR messaging or unscheduled and uncompensated phone calls).

The lack of integration of medical assistants (MAs) in the video visit workflow, resulting in a lack of previsit charting and medication reconciliation, was another concern. Incorporating MAs, or even involving the patient in their own previsit preparation, was deemed necessary for video visits to be sustained.

I'd like to see it be more efficient… In advance of the visit, it would be nice if they [patients] could do their medication reconciliation. …enter in the last set of vitals that they took for themselves. …enter in their own review of symptoms and chief complaints.

MD16

### Appropriateness: Suitability, Usefulness, and Practicability of Video Visits

Three themes emerged related to appropriateness of video visits: benefits and barriers, physical examination, and continuity of care, each of which are described below.

#### Benefits and Barriers of Video Visits for Patients and Families/Caregivers

Clinicians highlighted several benefits of video visits for patients and families/caregivers, including convenience, impact on travel and cost, and seeing the patient’s home environment. Barriers noted included access to technology, patient’s technological capacity, and language. Video visits were considered advantageous and convenient, saving patients time and money, particularly for older adults and those who travel long distances for appointments. Survey respondents (n=37; 77%) agreed that saving patients unnecessary travel was one of the top 3 benefits (Table 4). Several clinicians supported referring patients to local laboratories to further avoid unnecessary travel. Clinicians also noted that for patients who required assistance (eg, patients with dementia, epilepsy, or mobility issues), the video visit alleviated the travel burden on families/caregivers.

…it's very convenient for our patients, especially those who are elderly or have neurologic issues. [It] Spares them unnecessary travel and costs...

MD16

**Table 4:** Ambulatory neurology clinicians’ (n=48) top 3 areas of excitement related to video visits as reported through survey data.

With regard to video visits, clinicians (n=48) were excited about... (indicated top 3)	Survey respondents, n (%)
Saving patients from unnecessary travel	37 (77)
Increased access for vulnerable populations	33 (69)
Ability to see my patients from my home or nonclinic location	23 (48)
Reduced uncompensated work	18 (38)
Flexible scheduling of patient visits	17 (35)
Ability to see patients in their home environment	3 (6)
Ability to connect with patients’ caregivers/family members	3 (6)
Other (providers listed various reasons)	4 (8)

Several clinicians saw value in seeing patients in their home. Not only did it allow more family involvement, but clinicians could also troubleshoot daily functioning issues or modify the patient’s environment by directly assessing fall hazards, medications, and home devices.

…maybe you see … a lot of stuff strewn over the floor and that is why they're falling so much … you might observe things … that might impact their neurological disease on a day to day basis … or you don't think to ask about … in a clinic visit because you're not seeing the world that they're actually living in.

MD25

Even though increased access to vulnerable populations was rated as one of the top 3 benefits of video visits (n=33; 69%; Table 4), numerous clinicians mentioned that some patients, particularly older adults and lower-income patient populations (eg, unhoused individuals or rural farm workers), lacked the necessary access to technology and technological capability to support a video call. In the absence of a supportive family member/caregiver, video visits with patients with cognitive, hearing, or visual impairment were also considered nonideal.

... not suited are patients with some mild to moderate cognitive impairment. ...they have more trouble interacting with the physician and understanding who they’re talking to.

MD25

Language barriers were also considered a possible limitation of video visit utilization by both interviewees and survey respondents. Specifically, 17 (35%) surveyed clinicians indicated inclusion of interpreters was one of their top 3 concerns (Table 3).

#### Virtual Physical Examinations

Clinicians reported that video visits were superior to a phone call, allowing them to gather more information than just a medical history. Although exam needs varied by clinician and subspecialty, several clinicians described that despite challenges, they were pleasantly surprised to be able to perform several modified physical examinations over video. Nevertheless, many stated that the inability to perform a hands-on physical examination was a limitation of video visits that was best paired with a timely in-person follow-up. Several clinicians mentioned that virtual exams were more time-consuming and occasionally required assistance from a caregiver to position the phone to properly observe the patient, perform certain physician-directed exams, confirm what the patient says, or catch the patient if s/he is at risk of falling.

You have to have a caregiver, you can’t check gait without it [a caregiver] or the patient needs some sort of stand or desk or something where they can put the computer down at the end of a hallway and then asking the patient to find a hallway that they can walk back and forth is really the best way to do it. But a caregiver probably works best.

MD21

Additional limitations were related to the patient’s immediate environment, including adequate space for the patient to move around, and sufficient lighting. Camera positioning was also critical. If positioned too close, the clinician lacked sufficient visual field to observe the patient’s entire body. Clinicians also noted that occasionally patients took their video call at inappropriate times (eg, while working or driving), despite previsit counseling.

#### Promoting Continuity of Care

Clinicians considered video visits beneficial to ensure continuity of care for chronic conditions. There was general agreement that video visits are best suited for established patients, especially those who are stable/uncomplicated, or for a quick checkup without extensive examination or testing. Although several clinicians felt that most patients were appropriate for video visits, the majority agreed that new patient visits, and patients with acute conditions and declining health, were less suited due to relative ease of the complete physical examination when conducted in person. Many preferred to first see the patient in person before determining whether further follow-up care can be provided, at least partly, over video. Clinicians also recognized that some patient populations encounter significant barriers to attending the clinic in person and that video offers an opportunity to continue care.

We deal with progressive conditions, and once you get into that moderate to advanced stage of illness it just becomes not worth their effort to come anymore. It’s too difficult on the patient, it’s too difficult on the family, and so we lose contact with the vast majority of our patients as they become more advanced. ...telemedicine actually might allow continuity of care into the more advanced stages…

MD12

### Perceived Sustainability

Most clinicians were positive toward video visits and believed they could incorporate video into their practice long-term, although several insisted that video cannot replace a full in-person examination. Moreover, 40 (83%) surveyed clinicians agreed or strongly agreed that video visits supported their overall well-being. The general view was that for long-term sustainability, patient video visits will need to be selected carefully to optimize care and respect preferences. Patients’ suitability for video visits would need to be determined during scheduling based on several criteria (eg, physical examination needs, patient’s technological capacity and demographics, new versus return). However, previsit screening and triage to determine which patients are best suited for video was considered burdensome, which could compromise their sustainability. Seldom was the video visit itself considered a good tool for triage.

I would like to be able to offer it for my patients who are a little less mobile and who come from far away. I don’t think it will completely replace in-person visits, but if we can alternate visits and do in-person versus telehealth, I think that’ll be huge.

MD21

Most survey respondents (n=39; 81%) agreed that video visits should be supplemented with in-person visits. The recommended frequency of supplemental in-person visits varied among the respondents: 6 (15%) recommended quarterly, 11 (28%) biannually, 17 (44%) annually, and 5 (13%) every 2 years. A concern mentioned by a minority of interviewees and 7 (15%) surveyed clinicians was that patients may find video too convenient and opt out of recommended in-person visits. In contrast, 9 (19%) survey respondents with representation from epilepsy, memory, headache, stroke, and neuro-oncology, indicated that an all-video practice would be feasible for their patient population.

## Discussion

### Principal Findings

Video visits were rolled out rapidly at Stanford University’s Department of Neurology and Neurological Sciences during the COVID-19 pandemic. A high proportion of visits were conducted via video at volumes near prepandemic volumes, indicating robust and rapid adoption by both clinicians and patients. This necessary and widespread adoption allowed for thorough assessment of new opportunities and barriers in video-based care across almost all ambulatory neurology subspecialties.

Clinicians recognized the patient benefits of video visits, including saving patients unnecessary travel and increasing access to vulnerable populations, as is well-documented [1,3-8]. As patients with chronic progressive neurologic conditions lose function, in-person visits become increasingly challenging and care by video is a welcome new supplemental resource. Video visits could improve the experience for these patients and others, but various challenges remain. As clinicians noted, the most vulnerable high-need patients were often the hardest to care for virtually. Vulnerability is not uniform across patients and could be related to diagnosis (eg, patients with sensory or cognitive deficits), support (eg, caregiver status), socioeconomic status, or access (eg, poor access to technology). New creative solutions are being explored to address these challenges at various levels, with the goal of rendering virtual care an effective catchment for all. For instance, a pilot program delivering tablets to patients cared for by the US Department of Veterans Affairs has been successful in addressing concerns related to diagnosis and technology [40]. Further, municipality-level internet and broadband for all could address access issues [41].

Clinicians reported that video visits were beneficial for seeing the patient’s home and meeting caregivers and family members. Observing patients at home is a documented benefit of video visits; it can reduce patient and caregiver anxiety, and allow assessment of fall hazards and habitual behaviors that are not always possible to capture during an in-person visit [6,42]. These benefits were rarely identified within the top 3 benefits of video visits in the survey, which is likely related to the many perceived benefits of video visits as well as the diverse needs of neurology subspecialties’ patient populations. Interviewed clinicians emphasized that the presence of a caregiver is often essential to a successful video visit. For example, a caregiver is essential for collateral history when the patient has cognitive impairment or for safely examining gait for a complaint of parkinsonism, but not for a younger patient presenting with headache. Communicating the importance of including caregivers in certain video visits is thus important for optimal care provision.

Previously, the perceived limitations of virtual physical examinations have been a source of hesitant incorporation of telemedicine into neurology practice [10-12]; however, virtual physical examinations are possible, especially to supplement heavily history-based presentations [1,6,43]. A previous investigation demonstrated that virtual exams were adequate even for many patients with multiple sclerosis and neuroimmunology issues [44]. Presence of a caregiver can further facilitate a detailed and complete medical history and examination [28]. In this study, several clinicians reported that video cannot replace a full in-person examination, but others reported that the virtual physical examination was more feasible than expected. Best practices that were followed across clinics included consenting patients and explaining video limitations prior to the visit, and scheduling patients for in-person follow-up as soon as they are able to do so safely whenever an in-person examination was deemed important during the video visit. As clinics adjust to new routines and workflows, measures are also being taken to further optimize the virtual exam experience. For instance, the department has organized teleneurology professional development webinars on optimal virtual physical examination techniques [23,45], and guidance documents on this topic have also been developed internally. Together with time and experience, these efforts may further increase clinician comfort with the virtual physical examination.

Clinicians were keen about the ability to work remotely, reduction in uncompensated work, and flexible scheduling facilitated by video visit use. In the current configuration, video visits are not constrained by clinic spaces nor specific staffing needs, thereby allowing clinicians flexibility to vary timing and length of both their overall clinic session and individual patient appointments. This is a clear benefit, as lack of flexibility in work is a key driver of burnout [46], and even more salient given increased family and childcare duties for many due to the pandemic. Furthermore, reducing uncompensated work has potential well-being benefits. Historically, clinicians with full clinic schedules answered patient messages and phone calls uncompensated outside of normal clinical hours; this is another driver of physician burnout [47]. Video visits have relatively fewer constraints than in-person visits that are often booked out for months, and could enable the ability to address a new patient concern in short order “on the clock” and compensated. Further, most respondents also reported that video visits support their well-being, which is key in a specialty where an estimated 60% of clinicians reported at least one symptom of burnout even before the pandemic [48].

Perceived sustainability of video visits was high; clinicians emphasized a desire for video visits to remain a permanent fixture in their practice. However, the proportion of video visits and in-person visits within their practice was anticipated to change in their postpandemic practice and vary by individual providers and subspecialty. Most clinicians preferred to have a practice that uses both video and in-person visits, but more than half (57%) expressed a preference for less frequent in-person visits (ie, every 1 or 2 years). Perhaps most surprisingly, nearly 20% of clinicians from a variety of neurology subspecialties indicated that an all-virtual practice was possible. This variation in preferences may reflect the wide variation in the nature and examination demands of neurologic subspecialties, with subspecialties known to be especially exam-dependent (eg, movement and neuromuscular disorders) notably absent from those indicating feasibility of an entirely virtual practice.

The capabilities of mobile technology and remote monitoring have facilitated great advances in telemedicine [2,6,8,14]; however, several challenges remain and need to be addressed to enable long-term sustainability of video visits. Audio and video connectivity issues may be partially ameliorated by software updates and through increased experience with telemedicine, particularly for clinicians. On the other hand, some technological issues experienced by patients could potentially be addressed with targeted efforts, including more previsit planning and education with a staff member. To enhance the video visit experience for both patients and clinicians, pertinent features included virtual waiting rooms and multiuser interfaces to promote incorporation of trainees, interpretation services, and multidisciplinary care. Some of these features were added in subsequent updates and parallel workflows were developed to use other HIPAA-compliant platforms for visits including trainees or interpreters. In our experience, this parallel platform and approach were important for resolving some issues of functionality and service; however, the lack of integration and interoperability with standard EMR video functionality could impact sustainability.

Further analysis of the workflow around telemedicine in ambulatory neurology is needed as there is growing recognition that workflow is nontrivial and complex [49]. In this study, initial workflows for video visits did not include MAs taking patient vitals and performing medication reconciliation, key aspects of in-person visits for new patients. Clinic processes should reincorporate MAs in video previsits, perhaps through phone or video visits in the days prior to the clinical video visit. As clinical workflows evolve to sustain video visit use and meet the needs of the most vulnerable patient populations, these video previsits could potentially also provide patient education on optimal lighting, how to position devices, space, and presence of caregivers.

### Strengths and Limitations

Perceptions of clinicians using video visits were captured in real time during early rapid implementation through interviews and/or a survey across many ambulatory neurology subspecialties. However, given its nascent state, operational scheduling data only captured data of video visits conducted via the EPIC Hyperspace Platform and did not consistently reflect visits conducted on other HIPAA-compliant software, such as Zoom. Therefore, the data presented herein likely underestimate the actual total number of completed visits. Further, when video visits were diverted to phone calls or other software during the 8-week implementation period, the scheduling system incorrectly categorized the encounter as cancelled, no show, or patient left without being seen. This limitation has been recently addressed, presenting an opportunity to investigate the impact of video visits on clinic utilization in future investigations. Finally, views of other essential health care staff, such as patient care coordinators and MAs, who performed essential activities (eg, scheduling and confirming patients’ technology capabilities), as well as residents and patients/caregivers, are not presented here.

### Conclusions

Video visit adoption was rapid at Stanford’s ambulatory neurology clinics. Almost all clinicians conducted video visits by the eighth week of implementation and achieved near-normal patient volumes during the COVID-19 pandemic stay-at-home order. Despite the sudden change in workflow, clinicians largely expressed positive views toward video visits; clinicians supported permanent integration of video visits and noted them to be conducive to physician well-being. However, overall clinician satisfaction was impacted by technological issues, limitations with the physical examination, and challenges accessing vulnerable patient populations. Although our mixed methods evaluation confirmed the success of video visits in all subspecialty neurology clinics across many dimensions, innovations must be developed to address their limitations. Additional solutions are also needed for the most vulnerable patient populations. A crucial next step for optimization is to understand patients’ experiences and preferences.

## Appendix

Multimedia Appendix 1 Selected questions from 20-item neurology video visit clinician survey administered electronically via the REDCap survey tool.

Multimedia Appendix 2 Interview guide used to understand ambulatory neurology clinicians’ views on the acceptability, appropriateness, and sustainability of video visits in their practice.

## Abbreviations

APP: advanced practice provider
EMR: electronic medical record
HIPAA: Health Insurance Portability and Accountability Act
MA: medical assistant
