# Supplementary material for: Rapid Implementation of Video Visits in Neurology During COVID-19: Mixed Methods Evaluation
Source: J Med Internet Res. 2020 Dec 9;22(12):e24328. doi: 10.2196/24328 (PMC7732357; doi:10.2196/24328)
Supplement: Multimedia Appendix 1 [file jmir_v22i12e24328_app1.docx]

**Multimedia Appendix 1.** Selected questions from 20-item neurology video visit clinician survey administered electronically via the REDCap survey tool.

| **1.** | **On average, video visits should be supplemented by in-person visits at least every (choose one):^a^** | | | | |
| --- | --- | --- | --- | --- | --- |
|  | - 1. 3 months | | | | |
|  | - 1. 6 months | | | | |
|  | - 1. annually | | | | |
|  | - 1. every 2 years | | | | |
|  | - 1. unnecessary | | | | |
| **2.** | **What is your biggest concern regarding video visits at this time (pick up to 3)?^b^** | | | | |
|  | 1. Technological limitations (faulty audio/video)^c^ | | | | |
|  | 1. Being able to engage in training and education of residents and fellows^c^ | | | | |
|  | 1. Missing/losing the in-person connection/relationship with patients | | | | |
|  | 1. Including interpreters on video calls^d^ | | | | |
|  | 1. Difficulties arranging and completing necessary follow-ups after the video visit (e.g., scheduling follow-up appointments, labs, imaging, other testing) | | | | |
|  | 1. Insurance reimbursements for video visits are not the same as for in-person visits | | | | |
|  | 1. Patients unwillingness to come into clinic for requested in-person visits in the future^a^ | | | | |
|  | 1. Press-Ganey scores | | | | |
|  | 1. Patient expectations to have video visits as an option | | | | |
|  | 1. Maintaining access to readily available technology and equipment (e.g., laptop/desktop) needed for video visits | | | | |
|  | 1. Other (provider asked to describe in text box) | | | | |
| **3.** | **What are you most excited about video visits (pick up to 3)?^b^** | | | | |
|  | 1. Saving patients from unnecessary travel^d^ | | | | |
|  | 1. Increased access for vulnerable populations (end of life, remote, mobility limited) | | | | |
|  | 1. Ability to see patients from my home or non-clinic location | | | | |
|  | 1. Reduced uncompensated work (e.g., phone calls, MyHealth Messages) | | | | |
|  | 1. Flexible scheduling of patient visits | | | | |
|  | 1. Ability to see patients in their home environment^d^ | | | | |
|  | 1. Ability to connect with patients’ caregivers/family members^d^ | | | | |
|  | 1. Other (provider asked to describe in text box) | | | | |
| **4.** | **Having the ability to use video visits in my practice supports my overall wellbeing.^a^** | | | | |
|  | 1 – Strongly disagree | 2 – Disagree | 3 – Undecided | 4 – Agree | 5 – Strongly Agree |
| **5.** | **I anticipate being able to shift uncompensated work (phone calls/MyHealth messages) to compensated work via video visits.^c^** | | | | |
|  | 1 – Strongly disagree | 2 – Disagree | 3 – Undecided | 4 – Agree | 5 – Strongly Agree |

^a^ Sustainability, primary implementation outcome

^b^ Options were presented in random order for each participant

^c^ Acceptability, primary implementation outcome

^d^ Appropriateness, primary implementation outcome
