# Supplementary material for: Rapid Implementation of Video Visits in Neurology During COVID-19: Mixed Methods Evaluation
Source: J Med Internet Res. 2020 Dec 9;22(12):e24328. doi: 10.2196/24328 (PMC7732357; doi:10.2196/24328)
Supplement: Multimedia Appendix 2 [file jmir_v22i12e24328_app2.docx]

**Multimedia Appendix 2.** Interview guide used to understand ambulatory neurology clinicians’ views on the acceptability, appropriateness, and sustainability of video visits in their practice.

**INTERVIEWEE’S ROLE**

1. What is your role and which subspecialty are you a part of?
2. When did you start video visits and approximately how many have you done so far?

**VIDEO VISITS**

1. What has worked with video visits so far?
   1. What can you do in video visits that you would not otherwise be able to do?
2. What needs to change with video visits?
   1. What would you do differently?
3. What about video visits has been unexpected?
   1. Which parts of the video visits were better than you expected?
   2. Which parts of the video visits were worse than you expected?
4. What is the added value of video visits?
   1. For your team?
   2. For the patient and family/caregiver?
   3. For yourself?
   4. How does it impact the number of hours you work a day?
   5. How does it impact your interaction with the EMR?

**APPROPRIATENESS OF VIDEO VISITS FOR PATIENTS**

1. How do you determine a whether a patient is appropriate to be seen via video visit?
   1. What types of patients best suited for video visits?
   2. What types of patients are less suited for video visits?
      - demographics
      - family make-up/caregiver
      - diagnosis/severity
      - cognitive/communication limitations
      - technological sophistication
      - mobility/transportation limitations
      - team make-up
   3. Are there specific scenarios that you can think of where video visits are less appropriate?

**PHYSICAL EXAM INNOVATION IN VIDEO VISITS**

1. What are you unable to do in video visits that you wish you could?
   - - physical examinations
     - diagnostic tests
   1. How do you come around these challenges?
2. How have you been physical assessing patients over video?
   1. What are you doing differently that an in-person physical examination?

**SUSTAINABILITY**

1. In the short term, society will be opening up piecemeal. What are your priorities for seeing patients in person?
2. After the pandemic is over, how would you want to use video visits?

**RESIDENTS**

1. Have you been involved in setting up video visits with residents?
2. How have they been working out?

**TRAINING**

1. What kind of training did you receive in preparation for the video visits?
   1. Can you tell me more about that?
2. What kind of training would you have liked to receive?

**PATIENT EXPERIENCE**

1. What have patients reported their experience was?
2. Have you had any patients resist video visits? What was their reason?
3. How do video visits impact patient care?
4. How might video visits affect patient outcomes? How would you know?

**IMPACT OF COVID-19**

1. How has your workflow been impacted by COVID-19?
2. How has COVID-19 influenced your perceptions of video visits?

**WRAP UP**

1. Before we wrap up, is there something else that you would like to add?
